# MiR-148a-3p/SIRT7 Axis Relieves Inflammatory-Induced Endothelial Dysfunction

**DOI:** 10.3390/ijms25105087

**Published:** 2024-05-07

**Authors:** Camilla Anastasio, Isabella Donisi, Antonino Colloca, Nunzia D’Onofrio, Maria Luisa Balestrieri

**Affiliations:** Department of Precision Medicine, University of Campania Luigi Vanvitelli, Via L. De Crecchio 7, 80138 Naples, Italy; camilla.anastasio@unicampania.it (C.A.); isabella.donisi@unicampania.it (I.D.); antonino.colloca@studenti.unicampania.it (A.C.); marialuisa.balestrieri@unicampania.it (M.L.B.)

**Keywords:** miR-148a-3p, inflammation, endothelial dysfunction, mitochondrial damage, oxidative stress, apoptosis, SIRT7

## Abstract

In endothelial cells, miR-148a-3p is involved in several pathological pathways, including chronic inflammatory conditions. However, the molecular mechanism of miR-148a-3p in endothelial inflammatory states is, to date, not fully elucidated. To this end, we investigated the involvement of miR-148a-3p in mitochondrial dysfunction and cell death pathways in human aortic endothelial cells (teloHAECs) treated with interleukin-6 (IL-6), a major driver of vascular dysfunction. The results showed that during IL6-activated inflammatory pathways, including increased protein levels of sirtuin 7 (SIRT7) (*p* < 0.01), mitochondrial stress (*p* < 0.001), and apoptosis (*p* < 0.01), a decreased expression of miR-148a-3p was observed (*p* < 0.01). The employment of a miR-148a mimic counteracted the IL-6-induced cytokine release (*p* < 0.01) and apoptotic cell death (*p* < 0.01), and ameliorated mitochondria redox homeostasis and respiration (*p* < 0.01). The targeted relationship between miR-148a-3p and SIRT7 was predicted by a bioinformatics database analysis and validated via the dual-luciferase reporter assay. Mechanistically, miR-148a-3p targets the 3′ untranslated regions of SIRT7 mRNA, downregulating its expression (*p* < 0.01). Herein, these in vitro results support the role of the miR-148a-3p/SIRT7 axis in counteracting mitochondrial damage and apoptosis during endothelial inflammation, unveiling a novel target for future strategies to prevent endothelial dysfunction.

## 1. Introduction

MicroRNAs (miRNAs), as short noncoding single-stranded RNAs, interact with the 3′ untranslated region (3′ UTR) of target mRNAs, leading to inhibited translation or degradation [1]. These noncoding RNAs have emerged as crucial regulators in the pathogenesis of endothelial dysfunction and a key contributor to the development of diabetes and cardiovascular disorders, such as hypertension and atherosclerosis [2,3,4]. Therefore, the search for novel specific and sensitive biomarkers of endothelial dysfunction may have broader clinical significance. Different miRNAs display prognostic and diagnostic potential in inflammation-related endothelial dysfunction, given their involvement in the regulation of immune cells and inflammatory activity, targeting cytokines and modulating the transition of anti- and pro-inflammatory states of macrophages [5,6,7,8,9,10,11]. MiR-148a-3p, belonging to the miR-148/152 family [12], modulates endothelial cell function by affecting lipid metabolism and contributing to chronic syndromes [13]. MiR-148a-3p may oppose endothelial dysfunction by promoting proliferation and migration, and by inhibiting apoptosis via FOXO3 and FOXO4 [14], and it is able to attenuate inflammation, functional injury, and apoptosis in human umbilical vein endothelial cells (HUVECs) and macrophages [15,16,17]. Contrasting evidence reported the capacity of miR-148a-3p to promote M1 macrophage polarization and enhance inflammation via Notch signaling in septic mice [18]. Therefore, the exact role of miR-148a-3p in inflammatory endothelial dysfunction remains largely undefined, and the assessment of related molecular epigenetic pathways has not yet been conducted.

Emerging reports evidence the role of sirtuins (SIRTs) as epigenetic modulators acting on the progression and prognosis of inflammation [19,20,21]. In particular, among the seven sirtuins, the critical functions of SIRT1, SIRT6, and SIRT3 in preserving vascular function and endothelial inflammation have been extensively described [20,22,23,24,25,26,27,28]. SIRT4 was shown to prevent endothelial dysfunction and to repress inflammatory processes in response to cellular stress [19]. The downregulation of SIRT4 enhances pro-inflammatory cytokines and contributes to vascular damage [29]. Conversely, in endothelial models SIRT5 was demonstrated to promote thrombosis via the negative modulation of plasminogen activator inhibitor-1 (PAI-1) expression and to be involved in the ischemia-induced endothelial loss of the integrity of the blood–brain barrier, which determines occludin degradation [30,31]. In this context, the role of SIRT7 in endothelial damage has been poorly investigated.

SIRT7 overexpression reverted endothelial dysfunction and maintained cell homeostasis by regulating proliferation, migration, and tube formation in pulmonary arterial endothelium cells [32] and exerted a rescue effect against oxidative stress-induced HUVECs [33]. In response to inflammatory stimuli, SIRT7 promoted the activation of NF-κB signaling via Toll-like receptor 2, while its decay prevented the development of chronic inflammatory disorder [34]. Different studies reported an opposing role of SIRT7 during inflammatory stress, since sirtuin depletion was related to in vivo inflammation, as well as to attenuated inflammatory responses, barrier permeability, and endomesenchymal transition in human primary pulmonary endothelial cells [35]. Therefore, our understanding of the precise contribution of SIRT7 to endothelial dysfunction remains limited. In addition, although the ability of miRNAs to regulate SIRT7 expression has been described in different experimental models [33,36,37,38,39], to date, no report has explored the interaction between miR-148a-3p and SIRT7 in inflammatory-related endothelial dysfunction.

The present study was designed to investigate the in vitro biological function of miR-148a-3p and its molecular targets in endothelial cells during IL-6-mediated inflammation. 

## 2. Results

### 2.1. IL-6-Induced Inflammatory State

Among the many proinflammatory cytokines, the role of TNF-α has been well studied in the pathophysiology of cardiovascular diseases (CVDs) [40].

Interleukins (ILs), which are involved in inflammation, have also gained significant attention within the pathogenesis of CVDs, acting in a similar manner to that of TNF-α, thus leading to endothelial dysfunction [41]. 

Several studies already indicated that IL-6 exerts detrimental effects on vascular health through the activation of pro-inflammatory pathways and induction of oxidative stress [26,42,43].

To this end, we first confirmed the in vitro response of teloHAECs to pro-inflammatory cytokine IL-6, which is closely implicated during the onset of cardiovascular conditions, by inducing severe inflammatory stress [26,44,45]. TeloHAECs are immortalized human aortic endothelial cells with many of the cardinal features of endothelial cells, including angiogenesis potential, and a normal karyotype, which has been confirmed by cytogenetics and whole-genome DNA sequencing; thus, teloHAECs represent a good cellular model to study vascular endothelial cell activation [46]. In addition, as previously reported, the response to pro-inflammatory stimulation is highly concordant among immortalized teloHAECs, primary HCAECs, and HUVECs [47].

**Figure 1:**
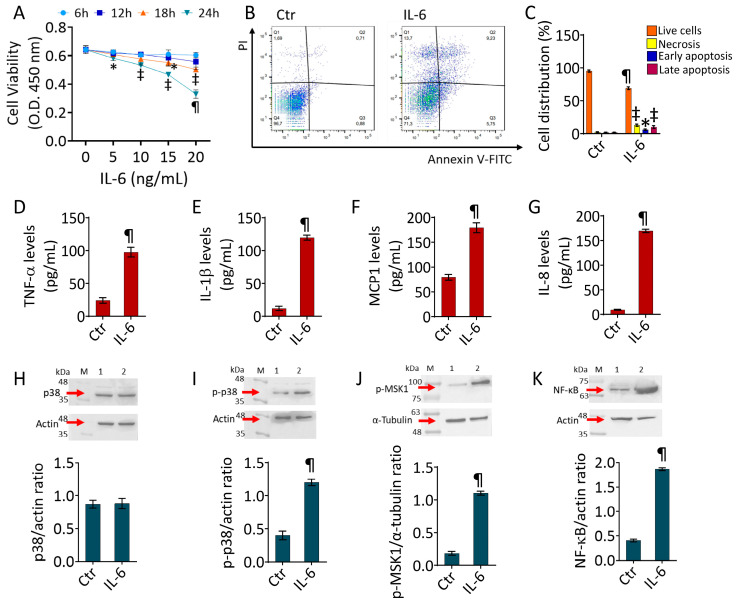
IL-6 induced apoptosis and inflammation. (**A**) Cell viability evaluated in teloHAECs exposed to different concentrations of IL-6 (5–20 ng/mL) up to 24 h. (**B**,**C**) FACS analysis of annexin V-FITC and PI-staining of teloHAECs treated for 24 h with 20 ng/mL IL-6 (IL-6). Evaluation of (**D**) TNF-α, (**E**) IL-1β, (**F**) MCP1, and (**G**) IL-8 content in teloHAECs treated with 20 ng/mL IL-6 for 24 h. Immunoblotting analysis of (**H**) p38, (**I**) p-p38, (**J**) p-MSK1, and (**K**) NF-κB protein levels in ECs treated with 20 ng/mL IL-6 for 24 h. Western blotting results are expressed as protein/housekeeping ratio. Control cells (0 ng/mL or Ctr) were maintained in complete culture medium with the corresponding volume of HBSS-10 mM Hepes. Q1, necrotic cells; Q2, late apoptotic cells; Q3, early apoptotic cells; Q4, viable cells. M, molecular weight markers, lane 1, Ctr, lane 2, IL-6. Data are expressed as the mean ± SD of n = 3 independent experiments. * *p* < 0.05 vs. 0 ng/mL or Ctr; ^‡^ *p* < 0.01 vs. 0 ng/mL or Ctr; ^¶^ *p* < 0.001 vs. 0 ng/mL or Ctr by Student’s *t*-test.

Dose–response experiments showed that exposure to IL-6 exerted cytotoxicity after 24 h of incubation with 20 ng/mL (*p* < 0.001 vs. Ctr) (Figure 1A). Therefore, the subsequent experiments were conducted by treating endothelial cells with 20 ng/mL of IL-6 for 24 h. Treatment with IL-6 caused the induction of cell death (Figure 1B,C). In detail, annexin /PI measurements revealed that IL-6 caused a reduction of live cells (71.3 ± 3.5% vs. 96.7 ± 2.4% in Ctr, *p* < 0.001) and increased necrotic (13.7 ± 2.9% vs. 1.69 ± 1.2% in Ctr, *p* < 0.01) and apoptotic (14.98 ± 2.7% vs. 1.59 ± 1.2% in Ctr, *p* < 0.01) populations (Figure 1B,C). As expected, IL-6 treatment promoted the release of cytokines in endothelial cells, as revealed by increased TNF-α, IL-1β, MCP1, and IL-8 content (*p* < 0.001 vs. Ctr) (Figure 1D–G). Moreover, the activation of the inflammatory p38/MSK1/NF-κB pathway was investigated using immunoblotting analysis (Figure 1). The results showed the ability of IL-6 to upregulate NF-κB, along with the phosphorylated forms of p38 and MSK1 protein levels (*p* < 0.001 vs. Ctr) (Figure 1I–K). On the contrary, exposure to the proinflammatory cytokine did not significantly modulate the total p38 expression (Figure 1H).

### 2.2. IL-6 Triggers Oxidative Stress and Mitochondrial Alteration

**Figure 2:**
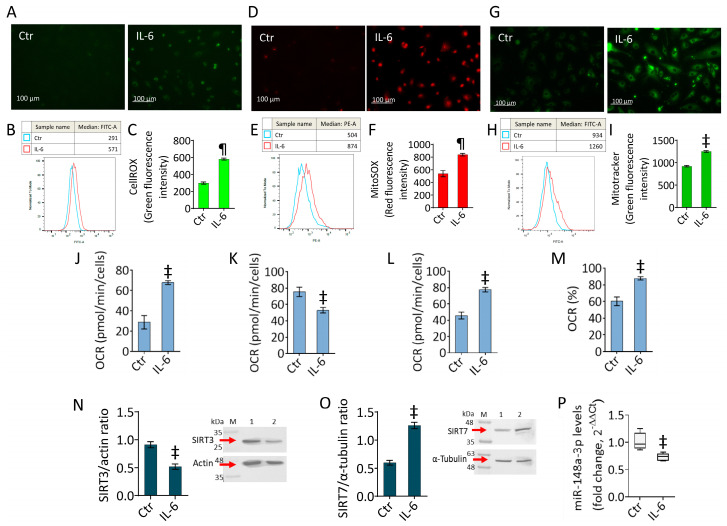
IL-6 effects on oxidative and mitochondrial state. Representative fluorescent images and cytofluorimetric analysis of (**A**–**C**) intracellular and (**D**–**F**) mitochondrial ROS content. (**G**–**I**) Representative images and FACS analysis of mitochondrial activity and assessment of (**J**) ATP production-coupled respiration, (**K**) maximal and (**L**) basal respiration, and (**M**) coupling efficiency evaluated using the Seahorse analyzer. Immunoblotting analysis of (**N**) SIRT3 and (**O**) SIRT7 protein levels, and (**P**) miR-148a-3p expression levels measured using qRT-PCR. The experiments were conducted on TeloHAECs treated with 20 ng/mL IL-6 for 24 h. Western blotting results are expressed as protein/ housekeeping ratio while miRNA levels are reported as floating bars with a line representing the median ± SD of n = 3 independent experiments. Control cells (Ctr) were maintained in complete culture medium with the corresponding volume of HBSS-10 mM Hepes. Scale bar, 100 μm. M, molecular weight markers, lane 1, Ctr, lane 2, IL-6. ^‡^ *p* < 0.01 vs. Ctr; ^¶^ *p* < 0.001 vs. Ctr by Student’s *t* test.

Given the crucial role of redox impairment during inflammatory response, the effects of IL-6 on the oxidative state of teloHAECs were evaluated. The results showed intracellular and mitochondrial ROS accumulation during IL-6 stimulation (*p* < 0.001 vs. Ctr) (Figure 2A–F). Based on the assessment of IL-6 cytotoxicity, the cytokine effects on mitochondrial function were investigated. The mitochondrial stain showed an increased fluorescent signal in IL-6-treated cells (*p* < 0.01 vs. Ctr), revealing mitochondrial activation (Figure 2G–I). When mitochondrial respiration and glycolytic state were assessed, the results showed that IL-6 upregulated ATP production-coupled respiration (*p* < 0.01 vs. Ctr), coupling efficiency (*p* < 0.01 vs. Ctr), and basal respiration (*p* < 0.01 vs. Ctr), while resulting in reduced maximal respiration (*p* < 0.01 vs. Ctr) (Figure 2J–M and Figure S1). The deleterious effect of IL-6 on mitochondrial function was also confirmed by the downregulated expression levels of SIRT3 (Figure 2N), the regulator of metabolic and antioxidant responses. According to our previous study, damaged mitochondria resulted in a SIRT3 downregulation [26]. Finally, the expression of miR-148a-3p and SIRT7 protein levels on the inflammatory state induced by IL-6 was then evaluated, given their emerging role during endothelial dysfunction [14,15,23,24,25]. The results revealed that treatment with IL-6 decreased miR-148a-3p expression (*p* < 0.01 vs. Ctr) while upregulating SIRT7 protein levels (*p* < 0.01 vs. Ctr) (Figure 2O,P).

### 2.3. MiR-148a Opposes the IL-6-Induced Inflammatory State

**Figure 3:**
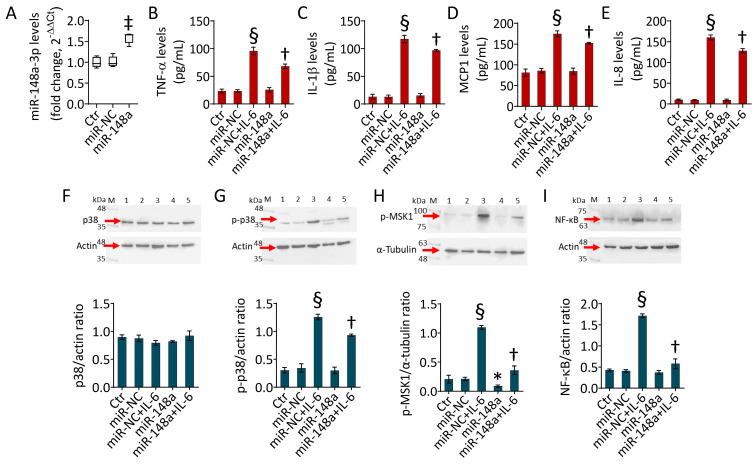
MiR-148a attenuated IL-6-related inflammation. (**A**) MiR-148a-3p levels measured using qRT-PCR in TeloHAECs transfected with mimic negative control (miR-NC) or hsa-miR-148a miRNA mimic (miR-148a). Assessment of (**B**) TNF-α, (**C**) IL-1β, (**D**) MCP1, and (**E**) IL-8 content using ELISA assays and immunoblotting analysis of (**F**) p38, (**G**) p-p38, (**H**) p-MSK1, and (**I**) NF-κB protein levels in TeloHAECs transfected with miR-NC or miR-148a before IL-6 treatment. Western blotting results are expressed as protein/housekeeping ratio, while miRNA levels are reported as floating bars with a line representing the median ± SD of n = 3 independent experiments. Control cells (Ctr) were maintained in complete culture medium with the corresponding highest volume of HBSS-10 mM Hepes. M, molecular weight markers; lane 1, Ctr; lane 2, miR-NC; lane 3, miR-NC + IL-6; lane 4, miR-148a; lane 5, miR-148a + IL-6. * *p* < 0.05 vs. miR-NC; ^‡^ *p* < 0.01 vs. miR-NC; ^§^ *p* < 0.001 vs. miR-NC; ^†^ *p* < 0.01 vs. miR-NC + IL-6 by one-way ANOVA with a Tukey’s post-test.

To test whether the establishment of the IL-6-induced inflammatory process was related to decreased miR-148a-3p expression levels, TeloHAECs were transfected with an hsa-miR-148a mimic (miR-148a) before cytokine stimulation (*p* < 0.01 vs. miR-NC) (Figure 3A) and the molecular mechanism was investigated. MiR-148a overexpression counteracted IL-6-induced cytotoxicity (*p* < 0.01 vs. miR-NC + IL-6) (Figure S2) and attenuated the release of proinflammatory cytokines, including TNF-α, IL-1β, MCP1, and IL-8 levels (*p* < 0.01 vs. miR-NC + IL-6) (Figure 3B–E). The immunoblotting analysis revealed that miR-148a overexpression significantly reduced p-p38, p-MSK1, and NF-κB protein expression (*p* < 0.01 vs. miR-NC + IL-6) (Figure 3F–I), indicating the ability of miR-148a to attenuate the inflammatory process.

### 2.4. MiR-148a Ameliorates Oxidative Stress and Apoptosis Caused by IL-6

**Figure 4:**
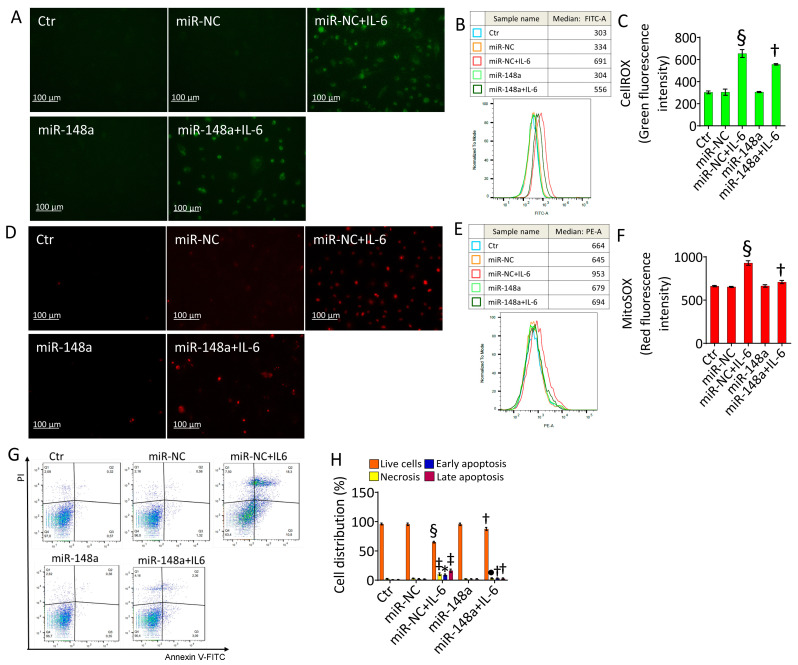
MiR-148a counteracted IL-6-induced oxidative stress and apoptosis. Representative fluorescent images and FACS detection of (**A**–**C**) intracellular and (**D**–**F**) mitochondrial ROS, and (**G**,**H**) dot plots and analysis of annexin V-FITC and PI-staining of TeloHAECs transfected with mimic negative control (miR-NC) or hsa-miR-148a miRNA mimic (miR-148a) before IL-6 treatment. Data are expressed as the mean ± SD of n = 3 independent experiments. Control cells (Ctr) were maintained in complete culture medium with the corresponding highest volume of HBSS-10 mM Hepes. Scale bar, 100 μm. Q1, necrotic cells; Q2, late apoptotic cells; Q3, early apoptotic cells; Q4, viable cells. * *p* < 0.05 vs. miR-NC; ^‡^ *p* < 0.01 vs. miR-NC; ^§^ *p* < 0.001 vs. miR-NC; ^•^ *p* < 0.05 vs. miR-NC + IL-6; ^†^ *p* < 0.01 vs. miR-NC + IL-6 by one-way ANOVA with a Tukey’s post-test.

The overexpression of miR-148a showed protective effects in TeloHAECs by ameliorating the IL-6-induced oxidative stress at both the intracellular and mitochondrial level (*p* < 0.01 vs. miR-NC + IL-6) (Figure 4A–F). In addition, miR-148a opposed live cell reduction (90.4 ± 2.7% vs. 63.4 ± 1.5% in miR-NC + IL-6, *p* < 0.01), as well as the increase of early (3.09 ± 1.1% vs. 10.8 ± 1.7% in miR-NC + IL-6, *p* < 0.05) and late (2.36 ± 0.7% vs. 18.3 ± 1.58% in miR-NC + IL-6, *p* < 0.01) apoptosis due to cytokine stimulation (Figure 4G,H).

### 2.5. MiR-148a Decreases the IL-6 Effects on Mitochondrial Damage

**Figure 5:**
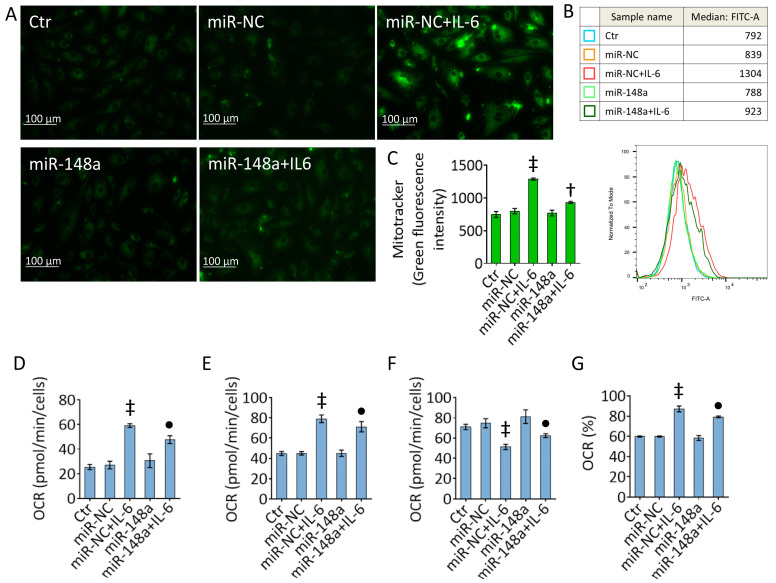
MiR-148a attenuated IL-6-related mitochondrial impairment. (**A**–**C**) Representative fluorescent images and FACS analysis of activated mitochondria and detection of (**D**) ATP production-coupled respiration, (**E**) basal and (**F**) maximal respiration, and (**G**) coupling efficiency evaluated using the Seahorse analyzer in TeloHAECs transfected with mimic negative control (miR-NC) or hsa-miR-148a miRNA mimic (miR-148a) before IL-6 treatment. Data are expressed as the mean ± SD of n = 3 independent experiments. Control cells (Ctr) were maintained in complete culture medium with the corresponding highest volume of HBSS-10 mM Hepes. Scale bar, 100 μm. ^‡^ *p* < 0.01 vs. miR-NC; ^•^ *p* < 0.05 vs. miR-NC + IL-6; ^†^ *p* < 0.01 vs. miR-NC + IL-6 by one-way ANOVA with a Tukey’s post-test.

The overexpression of miR-148a opposed the effects exerted by IL-6 on mitochondrial alterations (Figure 5 and Figure S2). In detail, miR-148a counteracted the IL-6-related mitochondrial activation (*p* < 0.01 vs. miR-NC + IL-6) (Figure 5A–C), reduced ATP production-coupled respiration, coupling efficiency, and basal respiration (*p* < 0.05 vs. miR-NC + IL-6), while increasing maximal respiration (*p* < 0.05 vs. miR-NC + IL-6) (Figure 5D–G and Figure S3). 

### 2.6. SIRT7 as a Target of miR-148a-3p

**Figure 6:**
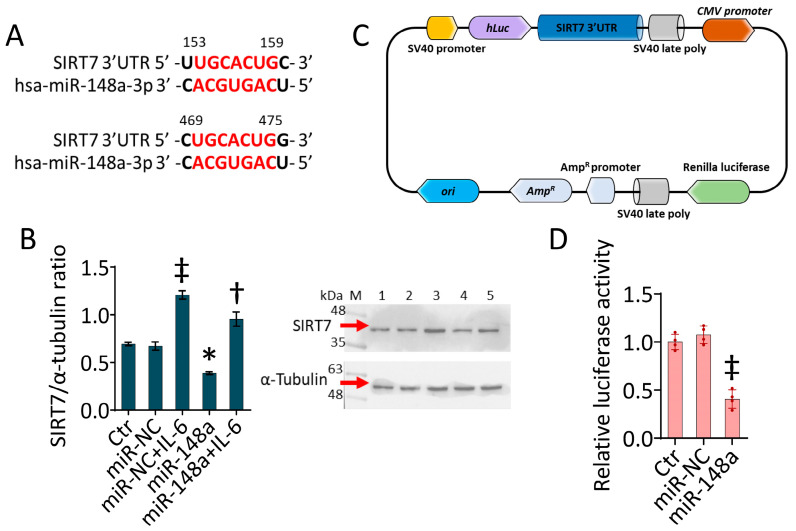
SIRT7 as miR-148a-3p target. (**A**) Alignment of hsa-miR-148a-3p with two regions (153–159 and 469–475) of SIRT7 3′UTR, identified using miRDB and TargetScan databases. (**B**) Immunoblotting analysis of SIRT7 protein levels in TeloHAECs transfected with mimic negative control (miR-NC) or hsa-miR-148a miRNA mimic (miR-148a) before IL-6 treatment. M, molecular weight markers; lane 1, Ctr; lane 2, miR-NC; lane 3, miR-NC + IL-6; lane 4, miR-148a; lane 5, miR-148a + IL-6. Western blotting results are expressed as protein/housekeeping ratio. (**C**) Schematic diagram of luciferase reporter plasmid containing the full 3ʹ-UTR region of SIRT7 gene. (**D**) Relative luciferase activity in TeloHAECs co-transfected with luciferase reporter plasmid and miR-NC or miR-148a. Data are expressed as the mean ± SD of n = 3 independent experiments. Control cells (Ctr) were maintained in complete culture medium with the corresponding highest volume of HBSS-10 mM Hepes. * *p* < 0.05 vs. miR-NC; ^‡^ *p* < 0.01 vs. miR-NC; ^†^ *p* < 0.01 vs. miR-NC + IL-6 by one-way ANOVA with a Tukey’s post-test.

A bioinformatic analysis revealed two predictive binding sequences for hsa-miR-148a-3p in the SIRT7-mRNA 3ʹ-UTR region (Figure 6A); therefore, functional studies were performed to ascertain whether SIRT7 could represent a target of miR-148a-3p in TeloHAECs during IL-6 stimulation. The immunoblotting analysis confirmed the induction of SIRT7 protein levels under IL-6 treatment (*p* < 0.01 vs. miR-NC) and revealed the inhibition by miR-148a overexpression (*p* < 0.05 vs. miR-NC), even in miR-148a + IL-6 cells (*p* < 0.01 vs miR-NC + IL-6) (Figure 6B), suggesting a possible relationship between miR-148a and SIRT7 in IL-6-stimulated cells. To provide the binding between SIRT7 and miR-148a-3p, a dual-luciferase reporter assay was performed, co-transfecting TeloHAECs with the luciferase vector containing the full 3′-UTR region of the SIRT7 gene (Figure 6C). Notably, miR-148a overexpression inhibited the luciferase activity of 3′-UTR SIRT7 (*p* < 0.01 vs. miR-NC) (Figure 6D), thus confirming SIRT7 as a target of hsa-miR-148a-3p.

## 3. Discussion

Herein, we report the first evidence on the protective role of miR-148a-3p in endothelial dysfunction by ameliorating the cytotoxic effects of the inflammatory pathways following exposure to IL-6. The results from this study show the ability of miR-148a-3p to attenuate endothelial cytotoxicity and to prevent inflammatory pathways, oxidative stress, mitochondrial alterations, apoptotic cell death, and SIRT7 upregulation under cytokine stress. These data suggest that miR-148a-3p, by targeting SIRT7, could represent a potential target in inflammatory responses within the endothelium. 

MiRNAs play a vital role in the functioning of endothelial cells. They have the remarkable ability to control the expression of genes, thereby influencing crucial cellular processes involved in endothelial inflammation, oxidative stress, and cell death [48].

Evidence showed that the development of a dysfunctional endothelium, which is a main driver of several chronic diseases, is closely related to different miRNAs and their regulatory mechanisms [48]. In septic models, miR-29b-3p overexpression alleviated lipopolysaccharide (LPS)-induced inflammatory responses in human coronary artery endothelial cells (HCAECs) [49], miR-483-5p and miR-6873-3p ameliorated endothelial injury in HUVECs exposed to oxidized LDL by activating autophagy and targeting TIMP2 and NF-κB, respectively [5,50], while exposure to IL-6 decreased miR-126-3p expression in the endothelial cell line EA. hy926, resulting in enhanced monocyte adhesion [51]. In this context, the detection of novel miRNAs as promising biological markers related to an impaired endothelium would be a useful prognostic and preventive strategy. Our data show that IL-6 decreased miR-148a-3p levels while inducing cytotoxicity and the release of inflammatory cytokines and proteins, such as NF-κB, and activated p38 and MSK1 in endothelial cells. Recent reports described the role of miR-148a-3p in endothelial dysfunction, although many mechanistic aspects remain unexplored. MiR-148a-3p was selectively upregulated in patients with atherosclerosis and its overexpression promoted proliferation and migration, while inhibiting apoptosis via FOXO4 and FOXO3 modulation [14], as well as counteracted the release of inflammatory factors in endothelial cells [15]. Accordingly, our data show that the restoration of miR-148a-3p expression by mimic transfection experiments attenuated the IL-6-induced cytotoxicity, inflammatory pathways, and apoptotic cell death in TeloHAECs. The relationship between p38, MSK1, and NF-kB pathways has already been reported during inflammation, as well as the effects of inhibitors or the knockdown of target genes [52,53]. Consistent with previous data reporting that upregulated miR-148a-3p reduced the expression of inflammatory mediators such as MSK1 and NF-κB in different experimental models [17,54], we found decreased NF-κB and activated MSK1 protein levels in miR-148a-transfected cells exposed to IL-6 stress. Our in vitro data suggest that MSK1 may serve as a noteworthy target of miR-148a-3p, resulting in a decrease in NF-kB. Undoubtedly, further studies using inhibitors or the knockdown of target genes could help to better clarify the role played by miR-148a-3p in endothelial inflammation pathways.

Upregulated miR-148a-3p also opposed the IL-6-induced cytokine pathways and apoptotic cell death, as previously described in THP-1 macrophages stimulated with oxidized LDL [16]. Recently, upregulated miR-148a-3p has been associated with reduced oxidative stress in angiotensin II-activated atrial fibroblasts, retinal epithelial cells exposed to hyperglycemia, and both in vivo and in vitro ischemia/reperfusion models [55,56,57]. Here, we show that IL-6 promoted intracellular and mitochondrial ROS accumulation in ECs, whilst miR-148a-3p overexpression attenuated both phenomena. Notably, we provide the first evidence of the effects of miR-148a-3p on mitochondrial respiration in endothelial cells exposed to IL-6 inflammatory stress. Physically, maximal respiration must be greater than or equal to basal respiration. However, our results showed that the maximal respiration in non-transfected ECs under IL-6 treatment, as well as in miR-NC + IL-6 and in miR-148a + IL-6 conditions, is lower than the corresponding basal respiration. We speculated that inefficient damaged mitochondria under IL-6 treatment in both naïve and transfected cells could require higher levels of ATP for maintaining organelle integrity, which could increase the basal oxygen consumption. Undoubtedly, as already reported, the persistence of unhealthy mitochondria impairs the mtDNA, which damages the integrity of the biogenesis program, leading to progressive damage in bioenergetic functions [58].

To date, studies on the modulatory role of miR-148a-3p on mitochondrial activity have only been reported on cancer models [59,60]. The beneficial effects mediated by miR-148a-3p might be related, at least in part, with its ability to regulate SIRT7 expression levels, although the intrinsic mechanisms underlying the role of SIRT7 on endothelial impairment are yet to be explored. A dual role of SIRT7 has been reported in both in vitro and in vivo models of the lung endothelium under LPS exposure [35]. Indeed, SIRT7 loss during LPS exposure has been associated with inflammation and fibrosis in murine lung tissues in vivo, while SIRT7 silencing suppressed LPS-induced pro-inflammatory responses and NF-κB signaling, by modulating the TGF-β pathway, in primary pulmonary endothelial cells [35]. The pulmonary endothelium-specific depletion of SIRT7 increased right ventricular systolic pressure and exacerbated right ventricular hypertrophy [32]. Moreover, in pulmonary arterial endothelium cells, the overexpression of SIRT7 reversed EC dysfunction occurring under pulmonary hypertension by deacetylating and stabilizing KLF4 [32]. Silencing SIRT7 in pulmonary artery or microvascular endothelial cells attenuates inflammation, induces endomesenchymal transition, and increases vascular permeability [35]. The beneficial effects of SIRT7 deprivation have been also described in acute kidney injury, where the sirtuin deficiency ameliorated the inflammatory response related to cisplatin treatment [61]. The activity of SIRT7 in endothelial dysfunction has been related to miRNA regulation [33]. Decreased SIRT7 and elevated miR-335-5p levels were found in HUVECs exposed to high glucose, TNF-α, and H_2_O_2_ levels, whilst sirtuin overexpression rescued endothelial function from oxidative stress and senescence [33]. To date, the functional relationship between miR-148a-3p and SIRT7 has only been described in hepatic lipid metabolism and hepatocarcinogenesis in mice [62]. Therefore, our data provide the first evidence of miR-148a-3p and SIRT7 interaction in this in vitro model, deepening knowledge of the molecular network involved in the vascular inflammatory state.

However, the role of SIRT7 in endothelial impairment and its contribution in inflammation require further investigations. The use of additional pre-clinical models would be needed for the deep evaluation of miR-148a-3p-based diagnostic or SIRT7-targeted therapeutic applications in inflammatory-related diseases, depending on the miR-148a-3p levels and the SIRT7 function on the cellular environment and other cell types, such as immune or epithelial cells. In addition, specific drug delivery and informatic platforms evaluating the therapeutic efficacy of miR-148a-3p ought to be developed; further, the identification of novel molecules/compounds targeting SIRT7 and the investigation of sirtuin downstream mechanisms are compelling necessities for therapeutics in the future.

## 4. Materials and Methods

### 4.1. Cell Growth, Treatment and Mimic Transfection

Endothelial cells from human aorta (TeloHAEC, CRL-4052, ATCC, Manassas, VA, USA) were grown in Vascular Cell Basal Medium (PCS-100-030, ATCC, Manassas, VA, USA) supplemented with Endothelial Cell Growth kit-VEGF (PCS-100-041, ATCC, Manassas, VA, USA). To mimic inflammatory state and determine oxidative stress, mitochondrial activation, and cell death, cells were treated up to 24h with different concentrations (5-20 ng/mL) of interleukin-6 (IL-6), as already described [26]. To induce overexpression of miR-148a, TeloHAECs were transfected with 20 nM hsa-miR-148a mimic (miR-148a, MCH01336, Applied Biological Materials, Inc. Richmond, BC, Canada) or mimic negative control (miR-NC, MCH00000, Applied Biological Materials, Inc. Richmond, BC, Canada) in medium without antibiotic and fetal bovine serum (FBS), using RNAifectin as transfectant reagent (G073, Applied Biological Materials, Inc. Richmond, BC, Canada). After 6h, FBS was added to the culture medium and maintained for 12 h before treatments. Control cells (Ctr) were maintained in complete culture medium with the corresponding highest volume of Hank’s balanced salt solution (HBSS)-10 mM Hepes.

### 4.2. Cell Viability

Cell viability was determined using the Cell Counting Kit-8 (CCK-8, Donjindo Molecular Technologies, Inc., Rockville, MD, USA). TeloHAECs were seeded in 96-well plates and, after treatments, were incubated at 37 °C with 10 μL CCK-8 solution for 4 h. Absorbance (450 nm) was detected with a microplate reader (model 680, Bio-Rad, Hercules, CA, USA).

### 4.3. Quantitative Real-Time PCR

Isolation of miRNAs and quantitative RT-PCR were performed as already described [45]. ID3EAL Individual miRNA RT Primer 1-plex (1103111-HSA0000243A, MiRXES, Singapore) and ID3EAL miRNA qPCR (1104101-HSA0000243A, MiRXES, Singapore) primers were used to quantify hsa-miR-148a-3p expression, and analysis was conducted following the 2−ΔΔCt method [63].

### 4.4. Cytokine Levels 

The levels of inflammatory mediators (TNF-α, IL-1β, IL-8 and MCP1) were assessed in culture medium using ELISA assays (Human TNF alpha, ab181421, Abcam, Cambridge, UK; Human IL-1 beta, ab214025, Abcam, Cambridge, UK; Human IL-8, ab214030, Abcam, Cambridge, UK; Monocyte Chemotactic Protein-1 Human, RAF081R, BioVendor, Brno, Czech Republic), as previously reported [26]. The absorbance at 450 nm was measured with a microplate reader (model 680, Bio-Rad, Hercules, CA, USA) and cytokine concentration was determined using the standard curve method.

### 4.5. Oxidative Stress Detection

Intracellular and mitochondrial ROS levels were evaluated by CellROX Green Reagent (C10444, Invitrogen, Waltham, MA, USA) and MitoSOX Red Mitochondrial Superoxide Indicator (M36008, Invitrogen, Waltham, MA, USA), respectively. Cells were stained with 5 μM fluorescent probes for 30 min at 37 °C. Images were recorded using the fluorescence microscope EVOS M5000 (Thermo Scientific, Rockford, IL, USA) and, after trypsinization, fluorescent signals were quantified using the FACS CANTO II flow cytometer (BD Biosciences, San Jose, CA, USA), collecting at least 10,000 events for each sample. Analyses were performed with FlowJo V10 software (FlowJo LLC, Ashland, OR, USA).

### 4.6. Mitochondrial Analysis

Mitochondrial state was assessed using MitoTracker Green FM (M7514, Invitrogen, Waltham, MA, USA), according to the manufacturer’s instructions. Cells were stained with 5 µM fluorescent probe for 30 min at 37 °C in the dark and imaged on a EVOS M5000 fluorescence microscope (Thermo Scientific, Rockford, IL, USA). Fluorescence intensity was measured using the FACS CANTO II cytometer (BD Biosciences, San José, CA, USA) and data was analyzed using FlowJo V10 software (FlowJo LLC, Ashland, OR, USA). 

Mitochondrial bioenergetics analysis was performed with Seahorse XF Cell Mito Stress Test Kit (103010-100, Agilent Technologies, Santa Clara, CA, USA) as previously described [64]. TeloHAECs were seeded in Seahorse assay microplate and oxygen consumption rate (OCR) and extracellular acidification rate (ECAR) were evaluated using XF HS Seahorse bioanalyzer (Agilent Technologies, Santa Clara, CA, USA). Oligomycin (1.5 μM), carbonyl cyanide-4(trifluoromethoxy) phenylhydrazone (FCCP) (1 μM), and rotenone/antimycin A (0.5 μM) were injected during the assay. 

### 4.7. Apoptotic Cell Death

Viable, necrotic, and apoptotic populations were estimated with FITC Annexin V Apoptosis detection kit (556547, BD Pharmigen, Franklin Lakes, NJ, USA), as previously reported [47]. After treatments, cells were detached by trypsinization and incubated in 500 μL binding buffer 1× with 2 μL Annexin V-FITC and 2 μL propidium iodide (PI) (20 μg/mL). Fluorescence intensity was quantified using FACS CANTO II flow cytometry (BD Biosciences, San José, CA, USA), recording at least 10,000 events for each sample, and analyses were conducted with FlowJo V10 software (FlowJo LLC, Ashland, OR, USA).

### 4.8. Protein Analysis by Immunoblotting

Cells were disrupted with lysis buffer and extracted proteins were separated as already described [63]. After nitrocellulose membrane transfer and non-specific binding blocking in 1× TBS 1% casein blocker (1610782, Bio-Rad, Hercules, CA, USA), membranes were incubated overnight at 4 °C with the following primary antibodies: anti-p38 (11,000, ab27986, Abcam, Cambridge, UK), anti-phospho-p38 (p-p38, 1,1000, ab32557, Abcam, Cambridge, UK), anti-phospho-mitogen- and stress-activated protein kinase-1 (p-MSK1, 11,000, 9591S, Cell Signaling Technology, Danvers, MA, USA), anti-Sirtuin 3 (SIRT3, 1500, Cell Signaling Technology, Danvers, MA, USA), anti-Sirtuin 7 (SIRT7, 1500, E-AB-60124, Elabscience Biotechnology Inc., Houston, TX, USA), anti-Nuclear factor kappaB (NF-κB, 11,000, ab16502, Abcam, Cambridge, UK), anti-α-tubulin (12,000, E-AB-20036, Elabscience Biotechnology Inc., Houston, TX, USA), and anti-actin (12,000, ab179467, Abcam, Cambridge, UK). After 1h of incubation with peroxidase-conjugated secondary antibodies, the chemiluminescent images were acquired with ECL substrate (E-IR-R301, Elabscience Biotechnology Inc., Houston, TX, USA) using the ChemiDoc Imaging System with Image Lab 6.0.1 software (Bio-Rad Laboratories, Milan, Italy). The density of protein bands was quantified using ImageJ software 1.52n version (Wayne Rasband, National Institutes of Health, Bethesda, MD, USA) and normalized to the relative loading control. 

### 4.9. Bioinformatics Analysis

Potential target genes for miR-148a-3p and the complementarity sites in the 3′ UTR of SIRT7 mRNA were estimated on miRDB, mirDIP, and TargetScan web servers. The mirDIP database (https://ophid.utoronto.ca/mirDIP/index.jsp#r) (Accessed on 4 October 2023) reported SIRT7 as a miR-148a-3p target with a top 5% (high) score class and 0.71 as an integrated score value. The miRDB database (https://mirdb.org/cgi-bin/target_detail.cgi?targetID=598181) (Accessed on 4 October 2023) identified two binding sites (153–159 and 469–475) in the SIRT7 3′ UTR, with 80 as the score value for hsa-miR-148a-3p. Bioinformatics analysis, carried out using TargetScan 8.0 (https://www.targetscan.org/vert_80/) (Accessed on 4 October 2023), confirmed both binding sites for miR-148a-3p in SIRT7 3′UTR. This prediction tool ascribed values of 86 as the context score percentile and −0.20 as the context score to the 153–159 region and a context score percentile value of 93 with −0.28 as context score for the 469–475 region.

### 4.10. Luciferase Assay 

The dual-luciferase pEZX-MT06 vector containing the full SIRT7 3′-UTR binding sequence (HMIT104110-MT06) and the miRNA Target clone control (CmiT000001-MT06) were purchased from GeneCopoeia (Rockville, MD, USA). Luciferase activity was detected using the Firefly and Renilla luciferase single tube assay kit (30081-T, Biotium, Fremont, CA, USA), as previously described [47]. 

### 4.11. Statistical Analysis

Statistical analyses were performed via multiple t tests by using GraphPad Prism 9.1.2 software (Software Inc., La Jolla, CA, USA). Data are represented as means ± standard deviations (SD) and statistical significance was set at *p* < 0.05.

## 5. Conclusions

In conclusion, the present study provides in vitro evidence of the protective role of miR-148a-3p on endothelial dysfunction. The overexpression of miR-148a-3p opposed inflammatory pathways, as well as oxidative stress, mitochondrial dysfunction, and apoptotic death in TeloHAECs exposed to IL-6. Notably, miR-148a-3p targeted the nucleolar SIRT7 protein levels. Overall, these results unveil the ability of miR-148a-3p to prevent endothelial damage, indicating miR-148a-3p/SIRT7 as epigenetic regulators to consider against endothelial dysfunction induced by inflammatory stimuli.

## Data Availability

The data that support the findings of this study are available from the corresponding author upon reasonable request.

## Supplementary Materials

The following supporting information can be downloaded at: https://www.mdpi.com/article/10.3390/ijms25105087/s1.
