# Supplementary material for: MiR-148a-3p/SIRT7 Axis Relieves Inflammatory-Induced Endothelial Dysfunction
Source: Int J Mol Sci. 2024 May 7;25(10):5087. doi: 10.3390/ijms25105087 (PMC11121049; doi:10.3390/ijms25105087)
Supplement: Supplementary file 1 [file ijms-25-05087-s001.zip › ijms-2980966-supplementary.pdf]

MiR-148a-3p/SIRT7 axis relieves inflammatory-induced endothelial dysfunction

Camilla Anastasio [1], Isabella Donisi [1], Antonino Colloca [1], Nunzia D'Onofrio [1,]* and Maria Luisa Balestrieri [1]

[1] Department of Precision Medicine, University of Campania Luigi Vanvitelli, Via L. De Crecchio 7, 80138 Naples, Italy; camilla.anastasio@unicampania.it (C.A.), isabella.donisi@unicampania.it (I.D.), antonino.colloca@studenti.unicampania.it (A.C.), marialuisa.balestrieri@unicampania.it (M.L.B.)

*Correspondence: nunzia.donofrio@unicampania.it (N.D.); Tel.: +39-081-566-7519

SUPPLEMENTARY MATERIALS

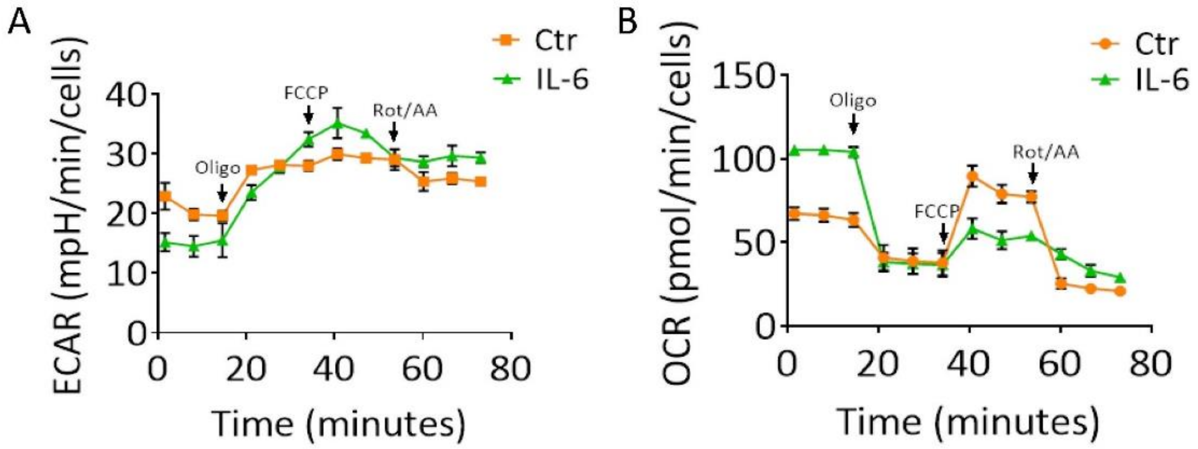

Figure S1. IL-6 effects on mitochondrial respiration. (A) Extracellular acetylation rate (ECAR) and (B) oxygen consumption rate (OCR) measured with Seahorse analyzer in TeloHAEC treated for 24h with 20 ng/mL IL-6 (IL-6) or maintained in complete culture medium with the corresponding volume of HBSS-10 mM Hepes (Ctr).

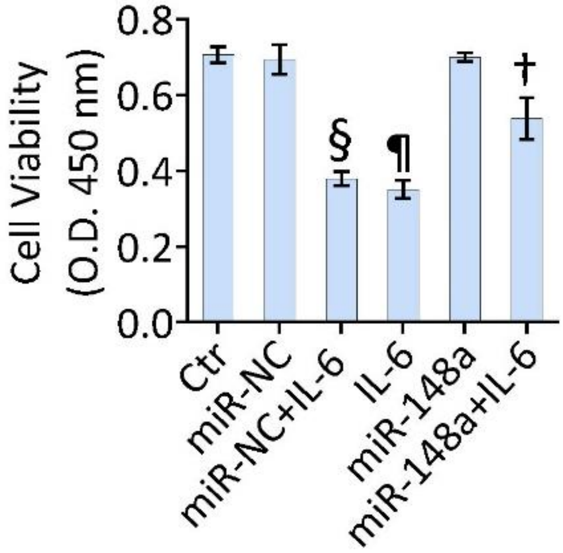

Figure S2. MiR-148a opposed the IL-6-induced cytotoxicity. Cell viability evaluated in TeloHAEC treated for 24h with 20 ng/mL IL-6 (IL-6) or transfected with mimic negative control (miR-NC) or hsa-miR-148a miRNA mimic (miR-148a) before exposure to IL-6. Control cells (Ctr) were maintained in complete culture medium with the corresponding highest volume of HBSS-10 mM Hepes. Data are expressed as the mean ± SD of n = 3 independent experiments. §$p<0.001$ vs. miR-NC; ¶$p<0.001$ vs. Ctr; †$p<0.01$ vs. miR-NC+IL-6 by one-way ANOVA with a Tukey's post-test.

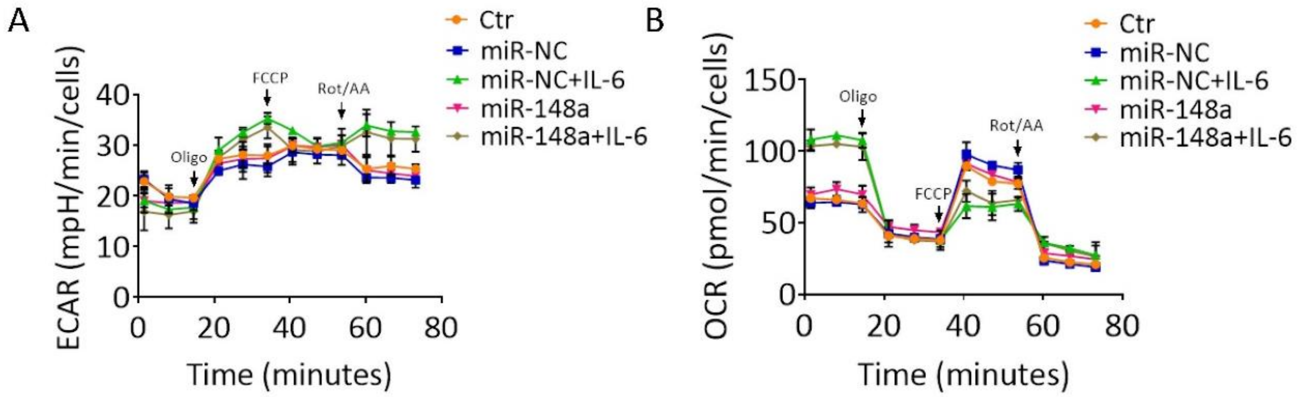

Figure S3. MiR-148a counteracted IL-6-induced mitochondrial dysfunction. (A) Extracellular acetylation rate (ECAR) and (B) oxygen consumption rate (OCR) measured with Seahorse analyzer in TeloHAEC transfected with mimic negative control (miR-NC) or hsa-miR-148a miRNA mimic (miR-148a) before IL-6 treatment. Control cells (Ctr) were maintained in complete culture medium with the corresponding highest volume of HBSS-10 mM Hepes.
